# Categorical Data in the Evaluation of School-Based Cyberbullying Prevention Programs: A Review of the Literature

**DOI:** 10.3390/bs16010093

**Published:** 2026-01-09

**Authors:** Andrés Antivilo-Bruna, Carmen Patino-Alonso

**Affiliations:** Department of Statistics, University of Salamanca, 37007 Salamanca, Spain; andres.felipe@usal.es

**Keywords:** categorical data analysis, contingency tables, cyberbullying prevention, school-based programs, logistic regression, systematic review

## Abstract

Categorical data analysis offers valuable tools for evaluating school-based prevention programs, yet these methods remain rarely applied in cyberbullying research. This literature review examined how categorical approaches, including contingency tables and related techniques, have been used in studies evaluating school-based cyberbullying prevention. A comprehensive search was conducted in Web of Science covering publications from 2020 to 2025, yielding 100 articles. After applying predefined inclusion and exclusion criteria, 24 studies were reviewed in full, of which 8 met all requirements for final analysis. The results revealed a predominant reliance on linear statistical techniques, such as *t*-tests, ANOVA, and regression models, applied mainly to continuous variables. By contrast, categorical analyses were seldom employed. The chi-square test appeared as the most frequent approach, but its use was generally restricted to descriptive purposes, with little application of complementary methods such as standardized residuals, effect size measures, or logistic models. This restricted application reduced the ability to capture response patterns, subgroup differences, and categorical associations essential for evaluating program outcomes. The findings highlight a methodological gap in cyberbullying prevention research and emphasize the potential of categorical data analysis to enrich interpretation. Incorporating these methods could increase methodological rigor, reveal nuanced behavioral patterns, and provide actionable evidence for educators, policymakers, and program designers seeking to strengthen school-based prevention strategies.

## 1. Introduction

### 1.1. Use of Categorical Data and Contingency Tables

The benefits of formulating statistical models to characterize the relationship between a dependent variable and one or more independent variables have been extensively discussed in the literature, and there are numerous methodological resources for characterizing this association when both, or at least one of the variables, are quantitative As early as 1971, Goodman highlighted the usefulness of stepwise regression procedures for selecting models that fit the data when both the dependent and independent variables are quantitative. Similarly, the linear regression model has proven to be a widely used alternative for analyzing how a set of variables (independent or explanatory) influence another (dependent or explained), allowing for the numerical estimation of the signs and magnitudes of the coefficients in a linear relationship (Gómez & Martínez, 2017). On the other hand, logistic regression models should be mentioned, a multivariate statistical technique that is used to predict a categorical dependent variable, which is usually dichotomous, through a set of independent variables that can be quantitative or qualitative. This same technique can also be used to estimate the probability of each of the possibilities of an event in more than two categories (Dávila & Ramírez, 2007). Finally, when the independent variable divides the data into two or more groups and the dependent variable is quantitative, one may turn to the broad family of Analysis of Variance (ANOVA) procedures, which allow for the examination of variation in a continuous response variable measured under conditions defined by discrete factors or classification variables (Dagnino, 2014).

None of the procedures described above are directly applicable when both variables are qualitative in nature, as occurs when attempting to predict the value of a dichotomous or polytomous variable based on another categorical variable (Goodman, 1971). Such qualitative data, measured on nominal or ordinal scales, typically arise from experiments or processes that can be classified into a finite number of mutually exclusive categories (Ramachandran, 2021). To study categorical variables simultaneously, specific techniques have been developed that analyze their association through a table displaying the joint distribution of absolute frequencies while retaining the original categories of each variable. This two-way table, known as a contingency table (CT), shows the number of cases corresponding to each combination of levels or factors of the variables involved. As Fienberg (2005) explains, in simple terms, a CT consists of a matrix of cells that present counts (whether of people, objects, or events), organized based on a cross-classification of two or more categories, which can adopt various sizes and structures. Its value lies in its capacity to reveal relationships between multiple categorical variables and to trace how those relationships evolve over time

Building on the study of frequency distributions within contingency tables, several statistical methods have been developed to assess the independence between the variables included in the table to be inspected through the application of probabilistic models. When dependence is observed, additional strategies enable the estimation of association strength using correlation coefficients such as Cramer’s V, Phi, Lambda, or the tetrachoric correlation coefficient, among others. Within this framework, Pearson’s chi-square goodness-of-fit test has become a fundamental nonparametric tool for analyzing categorical data, particularly in cases involving non-numerical variables or categorized quantitative variables. Furthermore, because it does not require the strict assumption of normality, the chi-square test is widely applied across diverse research fields (Valarmathi et al., 2024). Finally, chi-square test is also valuable for assessing intervention outcomes, as they allow researchers to determine whether statistically significant differences exist between groups before and after an intervention, using the chi-square test of independence for independent samples (Fisher et al., 2011).

However, when examining the type of information for which the chi-square test is typically applied, Osborn (1989) highlighted that, by itself, the test yields limited insight into data interpretation. In the same vein, the author noted that when used to analyze a contingency table, the chi-square test may fail to capture some of the most salient features of the data. Moreover, several authors have emphasized that its appropriate use requires reporting not only the chi-square value and its associated p-value, but also an accompanying measure of effect—such as the difference between two proportions, their ratio, or the odds ratio—along with the corresponding confidence interval (Fienberg, 2000; Osborn, 1989). At a minimum, it is recommended to compute and report standardized residuals based on the differences between observed and expected frequencies for each cell in the table (Pardo et al., 2010).

It is worth noting that contingency tables can be regarded as a method for examining relationships among a limited number of categorical variables—typically no more than three—and often serve as a preliminary step for more complex analyses of multidimensional relationships (López-Roldán & Fachelli, 2015). Although it is possible to identify in the literature on current categorical data the application of models and methods other than those just mentioned, such as log-linear and logistic models (Fienberg & Rinaldo, 2018), when more than two variables are incorporated into a CT, there does not seem to be a consensus among the scientific community on how to treat them. In other words, despite the efforts made in recent decades to establish unambiguous techniques for the analysis of CT, whether simple or multiple, there is still no robust theoretical framework that brings together all the possibilities observed in the applied field.

### 1.2. Cyberbullying and Prevention Programs

Traditional bullying, often manifested as harassment among classmates, has long been identified as a widespread but harmful experience with serious consequences for mental health and behavioral adjustment. In recent years, however, a distinct and increasingly prevalent form of aggression has emerged: cyberbullying, representing a growing concern due to its documented adverse effects on victims’ academic performance, psychological well-being, and overall mental health (Azami & Taremian, 2020). The profound and multifaceted impact of cyberbullying victimization on young people’s development- encompassing academic achievement, social functioning, and emotional resilience- has been widely substantiated in the literature. For instance, Batmaz et al. (2023) reported that cyberbullying exerts a direct and significant influence on both resilience and empathy. Moreover, heightened vulnerability to cyberbullying has been consistently observed among youth belonging to minority and marginalized groups, particularly in relation to gender and race/ethnicity (Amadori et al., 2025). Since the widespread adoption of the internet, access to and use of digital technologies have expanded exponentially, permeating nearly every aspect of daily life from education to recreation. Consequently, it has become increasingly difficult to identify contemporary activities or environments that are not, in some way, mediated by online interaction (Soriano-Molina et al., 2025).

Recent evidence indicates that while many characteristics of cyberbullying (such as its conceptualization, prevalence, risk and protective factors, consequences, and prevention strategies) are related to traditional bullying, it also exhibits unique features. The pervasive integration of digital technologies into young people’s lives enables perpetrators to intentionally and repeatedly harm others at any time, often anonymously and without immediate repercussions (Ansari, 2020). Digital media have therefore assumed a central role in adolescent social dynamics, contributing to the increased prevalence of risky behaviors such as cyberbullying and the spread of cyber-rumors (Bravo et al., 2022). As a result, cyberbullying has emerged as a growing concern across all school levels. Evidence suggests that bystander interventions, which train witnesses to respond constructively, can mitigate cyberbullying behaviors (Biernesser et al., 2023). Moreover, social-emotional skills play a critical role in this context, as difficulties with emotion regulation and empathy have been associated with both perpetrators and victims of cyberbullying.

Despite the advances outlined above, the socio-emotional processes underlying cyberbullying remain an active area of research, with some contradictory findings regarding the role of empathy in cyber victimization (Arató et al., 2020). Similarly, evidence remains inconclusive concerning the influence of schoolchildren’s moral reasoning and deterrence strategies on cyberbullying prevention (Lee & Jung, 2025). Current recommendations suggest that school-based interventions prioritize peer group norms, promoting positive peer influence and collective responsibility in online interactions (Lee & Jung, 2025). In addition, Arató et al. (2020) emphasize that, given that (i) both cyberbullies, and victims display lower empathic responsiveness and higher levels of moral disengagement compared with non-cyberbullies, and (ii) social-emotional skills are closely associated with cyberbullying behaviors, prevention programs should target the enhancement of emotional regulation and empathy among schoolchildren.

Furthermore, recent systematic reviews and meta-analyses indicate that outcomes related to the prevention and consequences of cyberbullying in schools vary substantially depending on the variables studied and the conceptual frameworks applied. The main findings and variables examined in reviews published since 2020 in the Web of Science, which focus on the characteristics of cyberbullying and effective intervention strategies, are summarized in Table 1.

As shown in Table 1, the recent literature includes studies that have systematically examined the characteristics of cyberbullying prevention interventions. Notably, Mula-Falcón and González (2023) emphasize that, despite the substantial increase in educational interventions targeting cyberbullying in recent years, there remains little conclusive evidence regarding their overall effectiveness. For instance, Ansari (2020) provides a detailed conceptualization of cyberbullying and its prevalence, including risk and protective factors, yet offers only a general overview of prevention and intervention programs. In contrast, Hajnal (2021), through a meta-analytic approach, provides more specific evidence, suggesting that programs incorporating socioemotional learning and mentoring are particularly effective in reducing cyberbullying perpetration. Nonetheless, these findings are not definitive. Torgal et al. (2023) concluded that the overall impact of school-based cyberbullying prevention programs remains statistically insignificant. Meanwhile, Kennedy et al. (2024) highlight how student characteristics (such as race, grade, and gender) affect victimization rates, underscoring the need to tailor prevention programs to the diverse needs of students. Finally, Li et al. (2024) report associations between child maltreatment, including emotional abuse and neglect, and both the perpetration and victimization of cyberbullying, emphasizing the importance of considering child maltreatment as a key risk factor when designing comprehensive prevention strategies.

In addition to providing evidence and recommendations regarding the factors most relevant in addressing cyberbullying within educational contexts, these studies also reveal a limited application of advanced techniques for analyzing categorical data. In this scenario, the present literature review aims to examine how statistical procedures for categorical variables are being implemented in studies primarily focused on cyberbullying prevention. By doing so, it seeks to provide substantive insights that enhance the interpretation of results in studies that, while conceptually sound, have not fully incorporated appropriate methods for categorical data analysis. Furthermore, this review aims to identify potential omissions or methodological inaccuracies in the application of these techniques, highlighting instances in which more precise or detailed reporting of results could have strengthened the evidence. The selection of studies for inclusion was guided by a set of predefined inclusion and exclusion criteria, alongside the search protocols described in the subsequent section. Finally, consistent with the objectives of the review, this work seeks to answer the following two research questions:

- Which data analysis strategies for categorical variables are reported in the school-based cyberbullying prevention programs and selected studies? Specifically, this includes the use of contingency tables, the chi-square test of independence and its associated p-value, correlational procedures, logistic regression models, and other relevant statistical techniques.
- To what extent do the strategies employed for categorical variables align with the methodological recommendations in the literature for the accurate interpretation of results? For example, do the studies report and interpret effect sizes, accompany the chi-square goodness-of-fit statistic with confidence intervals, or utilize standardized residuals, among other recommended practices?

## 2. Materials and Methods

In line with the objectives of this study, a review of the literature was conducted to examine the application of analytical techniques for categorical data in studies focused on cyberbullying prevention. This included both research evaluating the implementation of school-based prevention or prevention and intervention programs and studies aimed primarily at identifying substantive factors relevant to cyberbullying prevention. The review sought to assess the extent to which appropriate statistical analyses and interpretative practices were applied, as well as to identify potential omissions or misapplications. The entire process, in which both authors independently examined each record and report retrieved, was organized into three sequential phases: (i) development and execution of a comprehensive search strategy; (ii) selection of articles based on predefined inclusion and exclusion criteria; and (iii) coding of the selected manuscripts. The review was guided almost entirely by the guidelines recommended by PRISMA (Page et al., 2021), a strategy which facilitates data synthesis through a transparent and unbiased perspective. Indeed, as Oakley (2012) suggested, the review adopted an empirical approach that establishes explicit parameters for selecting primary data, aiming to produce a comprehensive overview of the evidence. Thus, the PRISMA 2020 checklist is presented in Table S1 (Supplementary Material). The whole process of search and selection of studies is openly available in the International prospective register of systematic reviews (ID: 1170871).

### 2.1. Eligibility Criteria and Search Strategy

Inclusion criteria were defined to guide the selection of studies pertinent to this literature review. The search focused on research addressing school-based cyberbullying prevention programs (whether exclusively preventive or combining prevention and intervention) or studies explicitly aimed at identifying variables relevant to the cyberbullying prevention within educational contexts. Eligible studies were required to be empirical, published in English or Spanish, and reported in peer-reviewed scientific journals. This notation referred exclusively to the language in which the manuscript was written, not to the participants’ language, as the inclusion criteria allowed studies conducted in any country and involving speakers of any language. They also needed to involve populations enrolled in primary or secondary education and, where applicable, include measures of outcomes achieved following program implementation. Studies were excluded if they were review articles (e.g., meta-analyses or systematic reviews), included populations outside the defined range (such as post-secondary students or programs targeting only preschool classrooms), appeared in non-academic publications, failed to meet the program criteria outlined above, or reported results irrelevant to the focus of this review.

An exhaustive literature search was conducted using the Web of Science database (Core Collection). For the search, only terms in English were used. The terms and syntax used for that database were entered as follows: cyberbullying AND (“prevention program*” OR “prevention AND intervention program*”). No additional search expanders or synonymous terms were applied. To focus the results, filters were applied to include only articles published in scientific journals between January 2020 and September 2025 (inclusive of both dates), in either English or Spanish. It is important to note that Spanish-language studies were included as Spanish-speaking countries make a substantive contribution to research on school-based cyberbullying prevention, and preliminary exploratory searches confirmed the availability of relevant empirical work in this linguistic context. This approach yielded a total of 100 articles, as summarized in Table 2. Web of Science was selected as the sole database because it provides broad, rigorous, and comprehensive coverage of peer-reviewed journals across psychology, education, and the social sciences, ensuring both the relevance and the quality of the included studies.

### 2.2. Inclusion and Exclusion Criteria

Only studies that explicitly reported the data analysis procedures employed to evaluate the implementation of cyberbullying prevention (and intervention, when applicable) programs in educational settings, as well as studies whose primary objective was to identify variables relevant to cyberbullying prevention, were selected. Studies were excluded if they did not report program implementation, focused solely on descriptive accounts of preventive strategies without incorporating statistical analyses of outcomes, or were not conducted in school settings. The detailed inclusion and exclusion criteria are presented in Table 3.

### 2.3. Article Selection Process

**Figure 1:**
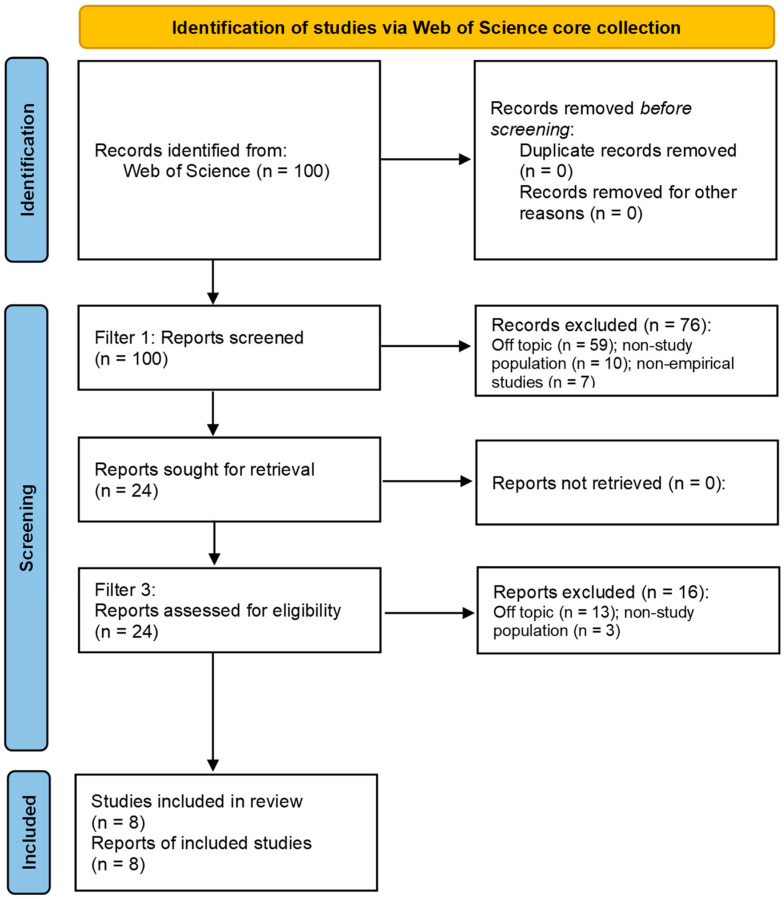
Flow diagram for the selection of articles for this review of the literature.

The search and selection process began with the identification of an initial pool of 100 articles. Of these, 8 were written in Spanish, and among the 8 studies that ultimately met all inclusion criteria, 3 were published in Spanish. This linguistic decision, while justified by the relevance of Spanish-language research in the field, may nonetheless have introduced a degree of selection bias, a point further elaborated in the Limitations section. The screening process proceeded through three sequential phases, applying predefined eligibility criteria at each stage, as depicted in Figure 1.

In the first phase, each article was screened based on its abstract. As the search was conducted exclusively within a single database (Web of Science), no duplicate records were identified. At this stage, the following inclusion criteria were verified: year of publication (2020–2025), language (English or Spanish), publication in a peer-reviewed scientific journal, empirical nature of the study, and explicit reporting of results related to prevention or combined prevention and intervention programs addressing cyberbullying within school contexts. Additionally, studies whose stated aim was to contribute to the development or improvement of cyberbullying prevention programs were also included. As a result, only those studies meeting all inclusion criteria were retained (n = 24).

In the second phase, full-text screening was performed. At this stage, it was verified that the selected articles provided sufficient information regarding the rationale for their chosen data analysis strategy, in accordance with each study’s stated objectives. Furthermore, the availability of the statistical data necessary to evaluate the potential use of contingency table analyses was assessed. Following this review, 8 studies were retained for final analysis, while the remaining 16 were excluded.

The studies finally selected for analysis were as follows: Buils et al. (2020), Cabrera et al. (2024), Escortell et al. (2023), Sorrentino et al. (2023), Touloupis and Athanasiades (2022), Williams et al. (2023), Wright et al. (2021) and Yurdakul and Ayhan (2023).

### 2.4. Coding of Variables

The selected manuscripts were reviewed and classified according to two main analytical axes: (i) the characteristics and relevance of the quantitative data analysis techniques employed to report the results derived from the implementation of cyberbullying prevention programs, and (ii) the type and quality of information provided to support the interpretation of results obtained from the analysis of categorical data. Table 4 presents both analytical axes and their corresponding categories.

Among the elements considered under Axis 1, the category “Techniques selected for data analysis and their methodological relevance” refers to the examination of the analytical techniques implemented in accordance with the objectives of each program. In particular, this category assesses whether the application of techniques associated with contingency tables (CTs) was feasible and whether their use contributed meaningful and interpretable information to the study. The category “Role of significant variables” aims to determine the number of variables evaluated within each program and to distinguish them according to the function they perform in the analytical model—either as independent and dependent variables, or as variables considered solely for descriptive or exploratory purposes. The category “Nature of significant variables”, in turn, was used to establish whether the variables were treated as categorical (dichotomous or polytomous) or continuous, a distinction that directly influences the choice of analytical strategy and the interpretation of the results obtained. Finally, the category “Omissions or limitations in the analytical process and interpretation of results” includes cases in which some essential analytical elements were not reported or where, despite being reported, they were not integrated into the interpretation of findings, thereby limiting the scope or validity of the conclusions drawn.

In contrast, Axis 2 focuses specifically on the analysis of categorical data, examining which statistical measures were most frequently reported (e.g., measures of independence, correlation coefficients), whether confidence intervals were included, whether effect size measures were presented and properly interpreted, and whether the information derived from standardized residuals was incorporated into the analytical process. This axis therefore provides a framework for evaluating the methodological robustness and transparency of the statistical procedures applied in studies addressing cyberbullying prevention.

## 3. Results

The presentation of results is organized according to the two analytical axes previously described. Each section provides detailed information on the specific characteristics of the eight studies that met the established selection criteria. Prior to presenting the findings, it is important to note that, in relation to cultural differences, most of the selected studies were conducted within European context. Specifically, 37.5% of the prevention programs analyzed were implemented in Spain (Buils et al., 2020; Cabrera et al., 2024; Escortell et al., 2023), follow by Greece (Touloupis & Athanasiades, 2022), Italy (Sorrentino et al., 2023) and Turkey (Yurdakul & Ayhan, 2023). A study conducted in the USA (Williams et al., 2023) has also been included, as well as another study that was carried out jointly in diverse cultural contexts (China, Cyprus, India and the USA) (Wright et al., 2021).

### 3.1. Axis 1: Characteristics and Relevance of Quantitative Data Analysis Technique

#### 3.1.1. Techniques Selected for Data Analysis and Their Methodological Relevance

As systematized in Table 5, there is considerable variability in the techniques selected to test the research hypotheses. Nevertheless, a pronounced tendency toward the use of linear techniques, characteristic of quantitative variable analysis, can be observed. Accordingly, tests for comparing means, such as the *t*-test or various ANOVA procedures, are frequently applied and generally employed to identify potential differences between pre- and post-implementation measurements of prevention programs. These techniques also structure their analyses by differentiating between independent variables (IVs) and dependent variables (DVs), which are generally quantitative in nature. It is noteworthy that these results are typically presented alongside an effect size measure, such as Cohen’s d, and others effect size are also reported when linear or logistic regression models are used for predictive purposes.

Regarding the use of techniques for categorical data, these appear to be employed far less frequently, with the chi-square statistic emerging as the only commonly used exception. Some of studies employ chi-square test as a preliminary step for more complex analyses, using it to evaluate whether significant differences exist in the proportion of subjects for dichotomous variables relevant to the study. For instance, results are reported for distributions according to sex (male-female) or the use of social platforms (yes-no), applicable both to the total sample and to specific subsets. To a lesser extent, chi-square is used to detect potential differences between control and experimental groups regarding other categorical variables.

#### 3.1.2. Role of Significant Variables

Two primary strategies for organizing variables within the selected studies were identified. The first, encompassing most of the studies that implemented prevention programs with school populations, aimed to assess the influence of a set of quantitative independent variables on a categorical dependent variable associated with cyberbullying—both in its negative forms (e.g., being a victim of bullying, coping with bullying, online victimization) and positive manifestations (e.g., protection against cyberbullying, emotional self-awareness). In this context, the most relevant variables were predominantly quantitative, as in many cases the sum of scores from Likert-scale instruments was employed, with the resulting total score serving as the reference value for understanding the impact of the implemented program.

The second strategy identified concentrated on examining associations between variables, predominantly through the application of correlation coefficients and regression models. In this context, the primary aim was to investigate the relationships between student involvement in cyberbullying behaviors (for instance, electronic harassment among peers) and psychosocial constructs such as empathy or perceived social support. These analyses provide insights into the magnitude and direction of these associations, rather than establishing causal linkages.

While both approaches offer valuable information regarding the interdependence of key variables underlying cyberbullying dynamics, the potential utility of categorical data analyses was largely overlooked. The integration of results could improve the depth and accuracy of interpretations, especially considering that the studies already included categorical variables.

#### 3.1.3. Nature of Significant Variables

Most of the studies analyzed employed variables measured at least at the interval level, a decision that plays a crucial role in how results are expressed and interpreted. Indeed, all reviewed studies incorporated at least two quantitative variables in their primary analyses. Notably, several studies based their findings on scales with graded response options; however, strategies were implemented to treat these ordinal data as though they reached the interval measurement level. Consequently, information on the distribution of responses for each item was often omitted, and only the mean scores corresponding to the overall dimensions were reported. Furthermore, when categorical variables were included, they generally served a descriptive rather than analytical function, with limited exploration of their potential influence on the observed relationships.

#### 3.1.4. Omissions or Limitations in the Analytical Process and Interpretation of Results

As previously noted, by prioritizing the quantitative properties of the data, none of the selected studies included frequency tables, contingency tables, or graphical representations (e.g., stacked bar charts) that could facilitate a clearer understanding of the results associated with categorical variables. It is important to recall that, in this context, frequency tables would enable the organization and summarization of response distributions for each scale item, thereby allowing for the identification of trends and distinctive response patterns that remain obscured when only the mean and standard deviation of the total dimension scores are reported. Likewise, the use and interpretation of total scores within a given dimension are debatable, particularly when such values are derived from the sum of responses on an ordered categorical scale (rather than actual scores), as is typical of instruments based on a Likert-type format.

Given these initial findings, it is not unexpected that the use of analytical procedures derived from the information provided by contingency tables is also quite limited, with their potential contribution generally confined to the descriptive characterization of the sample during the pre-intervention phase. In fact, except for the chi-square test, statistical techniques specifically designed for categorical variables are seldom employed to evaluate the effectiveness of prevention programs. The following section examines in greater detail how the chi-square test has been applied and whether the broader family of techniques associated with contingency table analysis has been effectively utilized.

### 3.2. Axis 2: Information Reported in the Analysis of Categorical Data

As shown in Table 5, the use of categorical data analysis techniques, whether univariate or bivariate, is limited. The latter, which would be ideal for group comparisons, was applied in only one study to examine differences between schoolchildren who participated in the prevention program and those in the control group. Although the strategy itself was appropriate, the results were reported solely using the chi-square value and its statistical significance, without including effect size measures, confidence intervals, or standardized residuals for each cell—information that would facilitate understanding whether the program had differential effects across groups. This omission is particularly notable, given that the study did report effect sizes when interpreting analyses of quantitative variables (e.g., Cohen’s d).

The application of logistic regression analysis, along with odds ratios, was also observed to evaluate the predictive value of variables relevant to cyberbullying prevention, taking into account the participants’ stage of adolescence. Unlike the previous study, all odds ratios in this case are reported alongside their corresponding 95% confidence intervals, providing a more complete and interpretable representation of the program effects.

Moreover, the chi-square test is somewhat more frequently used to assess the homogeneity of the sample with respect to categorical variables. This analysis is appropriate for establishing, prior to the implementation of prevention programs, the distribution of participants across variables such as gender or grade. However, it provides little substantive information regarding whether the program effectively achieves its intended outcomes. The rare cases where chi-square information was analyzed in depth occurred when that test was applied in the context of latent variables analysis, offering valuable insights for evaluating the goodness of fit of the tested model.

In summary, the application of categorical data techniques remains limited, generally confined to reporting the most common statistics without complementing them with effect size measures or detailed analyses of standardized residuals.

## 4. Discussion

The purpose of this review of the literature was to examine how statistical procedures for categorical variables are being implemented in studies addressing cyberbullying prevention. To achieve this objective, a comprehensive literature review was conducted, revealing an evident underutilization of analytical techniques specifically designed for categorical data, despite the clear potential of such approaches to enhance the understanding of mechanisms underlying cyberbullying prevention. The main finding indicates that most research in this field has relied predominantly on total scores derived from psychometric scales, subsequently analyzed through correlation coefficients, linear predictive models, analyses of variance, or structural equation modeling (SEM) (Peprah et al., 2024; Ma et al., 2024). While this methodological approach has allowed researchers to explore complex psychological processes and to model latent variables (thereby identifying the influence of certain factors on students’ beliefs and behavioral change), it also entails several methodological limitations. These include the following: (a) the assumption of metric properties in variables that are inherently categorical; (b) the loss of information regarding specific response patterns; and (c) the inability to precisely identify distinct behavioral differences across subgroups. Moreover, even when contingency tables are employed, the chi-square test is often applied in isolation, providing information only about the existence of a general association without indicating the specific categories in which the differences occur (McHugh, 2013). Indeed, its primary use within the reviewed studies was as a goodness-of-fit statistic, with limited application to the assessment of independence among two or more categorical variables. Although the chi-square test was the most widely applied categorical method, its use remained largely descriptive. Other analytical strategies, such as residual analysis, odds ratios, or logistic regression, were rarely incorporated, limiting the depth of interpretation.

It is important to recognize that CTs represent a robust methodological alternative for the analysis of categorical data, as they make it possible to examine associations between specific categories (e.g., type of cyberbullying behavior × gender or age). Unlike the use of aggregated or global scores, this analytical approach preserves the inherent complexity of the data rather than reducing it to a single summative value. In doing so, it facilitates the identification of excesses or deficits in frequency within each cell, thereby providing highly informative applied insights (Haberman, 1973; Beasley & Schumacker, 1995). This level of disaggregated analysis enables the identification of patterns of overrepresentation or underrepresentation within specific behavioral categories, which constitutes an analytical advantage that is particularly valuable for capturing the complex and multifaceted nature of cyberbullying.

A fundamental methodological contribution of this analytical approach lies in the use of standardized or adjusted residuals, which constitute the basis of post hoc procedures in contingency table analysis (Haberman, 1973). These residuals enable the precise identification of the specific category combinations that account for the overall association detected by the chi-square test, thereby preventing overly general or ambiguous interpretations (Beasley & Schumacker, 1995). Within the domain of cyberbullying research, this method allows for more nuanced insights—moving beyond the mere assertion of an association between variables such as gender and victimization to reveal the particular categories in which such differences are concentrated. In line with this cell-by-cell analytical perspective, research on school bullying has employed adjusted standardized residuals to detect over- and under-representations in specific categories of contingency tables, in some cases incorporating corrections for multiple comparisons to ensure statistical robustness (Romera et al., 2019; Favini et al., 2023).

Likewise, CT analysis facilitates the incorporation of effect size measures, representing another key methodological contribution. Whereas the chi-square statistics solely indicate whether an association is statistically significant, complementary indices such as the φ coefficient, Cramer’s V, or, in the case of 2 × 2 tables, the odds ratio (OR) and relative risk (RR), provide valuable information regarding the magnitude and practical relevance of the relationship between variables (McHugh, 2013). Furthermore, bias-correction procedures have been proposed for Cramer’s V when dealing with finite samples (Bergsma, 2013), thereby enhancing the accuracy and robustness of the inferences drawn from categorical data analyses.

Beyond the use of contingency tables, logistic regression—widely employed in recent research on cyberbullying—constitutes another essential technique for the analysis of categorical data, as it directly yields the odds ratio (OR) as an effect size measure while simultaneously allowing for the control of multiple covariates. Although this method does not provide the detailed, cell-by-cell perspective characteristic of contingency tables, it offers a probabilistic estimation of relative risk, thereby complementing frequency-based analyses. Studies such as that of Kunwar et al. (2024) exemplify the utility of this approach, as their regression models incorporate OR values to generate precise estimates of the likelihood of victimization or perpetration across different population groups.

Nevertheless, this study is not exempt from the limitations inherent to this type of review. The body of studies selected and analyzed reflects specific methodological choices—such as the exclusive use of a single database, the search terms employed, language restrictions, and the temporal scope—which may have resulted in the exclusion of potentially relevant research. In particular, relying exclusively on Web of Science may have constrained the scope of the evidence retrieved. We encourage future iterations of this line of research to consider extending the scope of the present work toward a more comprehensive systematic literature review. In doing so, subsequent studies could benefit from the inclusion of additional databases, such as Scopus, ERIC, or PsycINFO, thereby broadening the coverage of the evidence base and mitigating potential selection bias. Moreover, the coding scheme and analytical criteria employed, while systematic, may not have captured all relevant dimensions that could further strengthen the interpretation of the findings. To conclude this part, it is important to acknowledge that the methodological shortcomings identified in the studies included in this review may have shaped the conclusions reached. Inconsistent reporting of effect sizes, limited use of recommended analytical procedures for categorical data, and insufficient information regarding the verification of statistical assumptions restricted the comparability of methods and results across studies. Consequently, some of our interpretations may reflect the very constraints present in the studies reviewed. Therefore, enhancing and standardizing reporting practices in future research of this kind will be essential for improving the accuracy, robustness, and interpretability of subsequent literature reviews and systematic reviews.

## 5. Conclusions

In summary, this literature review highlights the critical underuse of statistical techniques adapted to categorical data in research on cyberbullying prevention. While conventional approaches based on total scores and linear models have advanced our understanding of program effects, they may obscure specific patterns of behavior and limit the detection of differences between subgroups. Analytical techniques for categorical data such as CTs, especially when combined with standardized residuals and effect size measures, offer a robust alternative for capturing detailed, cell-specific associations, allowing researchers to deepen their understanding of findings obtained through chi-square statistics. Similarly, logistic regression provides probabilistic estimates of relative risk while accounting for multiple covariates. More systematic integration of these categorical data methods could improve the accuracy, interpretability, and applied relevance of future studies on school cyberbullying prevention. Future prevention research should routinely integrate categorical data analysis to avoid overlooking subgroup-specific dynamics that are critical for effective program design.

## Figures and Tables

**Table 1:** Recent systematic reviews and meta-analyses on prevalence, impact, and school prevention programs for cyberbullying.

Authors (Year)	Studies Included	Main Variables Analyzed	Most Relevant Findings
Ansari (2020)	57	− Social media applications (Apps) − Risk and protective factors − Gender − Role of parents − Role of educators and schools − Role of peers − Role of health care providers − Specific laws	This review critically examines current knowledge on cyberbullying, including its definition, risk and protective factors, adverse psychosocial consequences, and the design and implementation of prevention and intervention programs
Hajnal (2021)	23	− Social-emotional learning − Comprehensive school approach − Mentoring − Online safety and cyberbullying education	Evidence suggests that school-based cyberbullying prevention programs integrating socioemotional learning and mentoring yield the greatest reductions in perpetration, while those focused on cyber safety and cyberbullying education are most successful in mitigating victimization.
Kennedy et al. (2024)	87	− Race or ethnicity − Grade (school level) − Gender − Study region/area − Factors related to survey design	Trends in victimization rates for both traditional bullying and cyberbullying fluctuate over time according to race, grade, and gender
Li et al. (2024)	57	− Child abuse − Cyberbullying − Sample characteristics − Type of publication − Research design	Experiences of child abuse, whether manifesting as neglect or emotional abuse, are linked to an increased likelihood of both cyberbullying perpetration and victimization.
Mula-Falcón and González (2023)	9	− Educational prevention programs − Levels of cyberbullying perpetration	Educational prevention programs could reduce levels of cyberbullying perpetration among schoolchildren aged 10 to 17.
Torgal et al. (2023)	9	This study examined the impact of school programs designed to prevent cyberbullying among students at the elementary and secondary levels	Although the overall effect of school-based cyberbullying prevention programs did not reach statistical significance, moderator analyses suggest that the inclusion of an empathy activation component enhances program effectiveness.

Source: Compiled and elaborated by the authors.

**Table 2:** Search terms and guidelines.

Syntax (in Article Title, Abstract or Keywords)	Cyberbullying AND (“Prevention Program*” OR “Prevention AND Intervention Program*”)
Databases	Web of Science (Core Collection)
Year	From January 2020 to September 2025
Country	All
Source	Scientific journals
Type	Article
Full text	Yes
Peer-reviewed	Yes
Total	n = 100

Source: Compiled and elaborated by the authors.

**Table 3:** Summary of inclusion and exclusion criteria applied.

Variable	Inclusion Criterion	Exclusion Criterion
Document type	Scientific articles	Other publications
Language	English or Spanish	Other languages
Access	Free access or available	Restricted access
Type of study	Studies reporting quantitative results of cyberbullying prevention programs	The prevention program was not implemented (e.g., theoretical or descriptive articles on the programs) or its results are not analyzed quantitatively
Setting	School context	Contexts outside of school
Level	Primary or secondary school students	Other educational levels or inclusion of the general population
Purpose of the study and characteristics	Evaluation of a prevention or prevention and intervention program (emphasizing the preventive element) Study that explicitly states	Studies evaluating programs unrelated to prevention
Participants	Program aimed only at schoolchildren	Programs focused on other members of the school community (e.g., primary caregivers of schoolchildren or teachers)

Source: Compiled and elaborated by the authors.

**Table 4:** Categories of analysis of the articles.

Axis 1: Characteristics and Relevance of Quantitative Data Analysis Technique	Axis 2: Information Reported in the Analysis of Categorical Data
Techniques selected for data analysis and their methodological relevance Role of significant variables Nature of significant variables Omissions or limitations in the analytical process and interpretation of results	Calculated statistics and corresponding confidence intervals Effect size measures Standardized residuals

Source: Compiled and elaborated by the authors.

**Table 5:** Summary of the characteristics of the selected data analysis techniques.

Authors, Year, and Country of Study	Type of Study	Statistical Techniques Applied	Nature and Role of Significant Variables
Buils et al. (2020), Spain	Quasi-experimental pre-post design with two groups	T-tests, chi-square, variance analyses (ANOVAs), covariance analyses and effect size are calculated by means of Cohen’s d statistic	Quantitative DV (scores): emotional self-awareness, problem solving, responsible use, digital teaching tutoring and family supervision Categorical IV: Program “Living in harmony in the real and digital world takes”
Cabrera et al. (2024), Spain	Cross-sectional correlational quantitative study	Chi-square, Pearson correlation coefficient, *t*-test, odd ratio and logistic regression analysis	Categorical DV: be a victim of cyberbullying Categorical IV: stages of adolescence, cybervictimization, pleasant cyberbullying emotions. Quantitative IV: family cohesion, experiencing family conflicts, perceived social support, physical ability, physical appearance, relationship with peers, relationship with parents, general self-concept, language self-concept and mathematical self-concept
Escortell et al. (2023), Spain	Cross-sectional correlational quantitative study	Chi-square, Latent Class Analysis and MANOVA,	Quantitative variables: electronic harassment behaviors among peers, learning goals, achievement goals and social reinforcement goals
Sorrentino et al. (2023), Italy	Longitudinal research design	Simple correlations with Cohen’s interpretation of r-values, hierarchical regression analysis (coefficients were reported with confidence intervals)	Quantitative variables: Participants’ involvement in cyberbullying and cybervictimization, Students’ involvement in school bullying and victimization, Empathy, Moral Disengagement, Increasing Self-Awareness of Cyberbullying, students’ perceived social support, Parental online monitoring strategies and School climate.
Touloupis and Athanasiades (2022), Greece	Experimental longitudinal research design	Confirmatory factor analysis (involving the report of chi-square), T-test, repeated measures ANOVA, MANOVA	Quantitative DVs: cyberbullying involvement (online victimization and online bullying). Categorical IV: preventive program TABBY Others: self-esteem
Williams et al. (2023), USA	Cluster-randomized comparison group design with pre-test and post-test surveys (schools were matched by geographical region and enrollment size)	GLM and multilevel analyses using mixed models, chi-square	Quantitative DVs: behavior and knowledge regarding physical, social, verbal, and cyberbullying victimization and perpetration; hypothesized risk and protective factors; and the skills, knowledge and attitudes targeted by the intervention. Categorical IV: LST prevention program with added bullying prevention content.
Wright et al. (2021), China, Cyprus, India and the USA	Longitudinal cross-cultural design	Multigroup confirmatory factor analysis, Multigroup structural equation model (this includes the use of the chi-square goodness-of-fit test) and Pearson correlation coefficient	Quantitative DV: Cyberbullying involvement (i.e., perpetration, victimization) Quantitative IV: peer attachment, social preference goals
Yurdakul and Ayhan (2023), Turkey	Quasi-experimental design, including intervention and control groups and pretest- post-test follow-up test	Wilcoxon Signed Rank Test	Categorical IV: Group (control or intervention) Quantitative DV: Cyberbullying tendency, Coping with cyberbullying, Protection from cyberbullying

Source: Compiled and elaborated by the authors.

## Data Availability

No new data were created or analyzed in this study. Data sharing is not applicable to this article.

## Supplementary Materials

The following supporting information can be downloaded at: https://www.mdpi.com/article/10.3390/bs16010093/s1, Table S1: PRISMA 2020 checklist for systematic reviews.
